# School and kindergarten attendance and home schooling of pediatric cancer patients before and during the SARS-CoV-2 pandemic: results of a survey of the German Society for Pediatric Oncology and Hematology

**DOI:** 10.3205/dgkh000381

**Published:** 2021-03-05

**Authors:** Arne Simon, Benjamin Siebald, Walther Stamm, Norbert Graf, Stephan Meier, Martin Schrappe, Andreas H. Groll, Hans-Jürgen Laws, Thomas Lehrnbecher

**Affiliations:** 1 Pediatric Hematology and Oncology, Children’s Hospital Medical Center, University Clinics, Homburg, Germany; 2 Division for Pediatric Hematology and Oncology, Hospital for Children and Adolescents, University Hospital, Goethe University Frankfurt am Main, Frankfurt, Germany; 3 Department of Pediatrics and Children’s Cancer Research Center, TUM School of Medicine, Technical University of Munich, Kinderklinik München Schwabing, Munich, Germany; 4 Rehabilitation Center for Pediatric Hematology and Oncology Katharinenhöhe gGmbH, Schönwald, Germany; 5 Pediatrics I, University Medical Center Schleswig-Holstein, Campus Kiel, Kiel, Germany; 6 Infectious Disease Research Program, Center for Bone Marrow Transplantation and Department of Pediatric Hematology/Oncology, University Children’s Hospital, Münster, Germany; 7 Department of Pediatric Oncology, Hematology and Clinical Immunology, University Hospital Düsseldorf, Düsseldorf, Germany

**Keywords:** children with cancer, infection prevention, SARS-CoV-2, school, day care center, home schooling

## Abstract

In this multicenter survey (July 07 to August 08, 2020) in pediatric oncology centers (POCs) belonging to the German Society for Pediatric Oncology and Hematology (GPOH), 36 POCs participated (response rate 70.6%). Home schooling practice was judged as satisfying by 79% prior to and by 38% during the pandemic (*P*=0.0007). The individual risk of a SARS-CoV-2 infection and the risk of transmission to other patients/caregivers were arguments against attendance. Most POCs recommended regular social participation/school attendance after the end of intensive therapy. 81% stated that persisting restrictions result in serious negative psychosocial consequences for the patients and their families. In-hospital school education, home schooling and re-attendance of school and kindergarten among pediatric cancer patients have suffered a severe setback during the SARS-CoV-2 pandemic. Continuous communication and education concerning protective measures as well as an individual risk assessment are required to avoid the detrimental exclusion of pediatric oncology patients from kindergarten and school.

## Zusammenfassung

An diesem multizentrischen Survey (7. Juli–8. August 2020) in pädiatrisch-onkologischen Zentren (POCs) der GPOH nahmen 36 POCs teil (Antwortrate 70,6%). Vor der Pandemie beurteilten 79%, während der Pandemie nur 38% den Hausunterricht als zufriedenstellend (*P*=.0007). Das individuelle Infektionsrisiko und das Risiko einer Übertragung auf andere Patienten oder medizinisches Personal waren die wichtigsten Argumente für soziale Distanzierung. Nach Ende der intensiven Therapie befürworten die meisten POCs einen regulären Schul- oder Kindergartenbesuch. 81% befürchten bei anhaltendem Ausschluss schwerwiegende psychosoziale Konsequenzen für die Patienten und ihre Familien. Die Auswirkungen der Pandemie auf die Edukation und soziale Teilhabe von Kindern mit Krebserkrankung sind gravierend. Kontinuierliche Information und Kommunikation über Präventionsmaßnahmen sowie eine individuelle Risikoanalyse sollten helfen, den Ausschluss während (Hausunterricht, Klinikschule) und nach (Kindergarten und Regelunterricht) einer Krebstherapie zu vermeiden.

## Background

The primary goal of treatment in pediatric cancer patients is a sustained remission of the underlying malignancy, with a minimum of treatment intensity to reduce short- and long-term adverse effects and impact on development and quality of life [1], [2], [3], [4], [5], [6]. However, intensive treatment in pediatric cancer patients may cause prolonged interruption of school or kindergarten attendance [7]. Therefore, most pediatric oncology centers (POCs) have established an interdisciplinary collaboration [8] in order to provide continuous education as far as possible [9], which, unfortunately, is not standardized across the centers. Although early re-entry to school or kindergarten is supported as a standard of care after the end of intensive treatment [10], [11], regular attendance of school during intensive treatment is usually not possible due to a variety of factors. To alleviate these restrictions, individual home schooling and in-hospital schooling by specialized teachers are important activities. In recent projects, internet-(video) based [12] school attendance or the use of avatars [13] have been tested to connect patients with their peers, which at least to some extent allows school-related participation and interaction. A recent UK report about educational provision for children unable to attend school for medical reasons came to the conclusion that without education, children and young people with medical conditions are disadvantaged and they, their families and society may continue to pay the financial and social costs for generations [14].

One argument against unrestricted school or kindergarten attendance during intensive treatment is the severe immunosuppression, which puts the patient at an increased risk of infectious complications [15]. However, studies examining the risk of severe infections in pediatric cancer patients related to kindergarten or school attendance are rare [16], [17], and do *not* support the assumption that rigorous social restrictions lead to lower infection rates. The SARS-CoV-2 pandemic and the fear of acquiring and transmitting this potentially devastating infection (COVID-19) [18], [19], [20], [21] have probably exacerbated the previously known challenges of kindergarten or school attendance in pediatric cancer patients [22], which may also impact their healthy siblings [23], [24], [25]. In addition, teachers responsible for home teaching or in-hospital teaching may allocate themselves to a COVID-19 risk group or may be reluctant to perform individual teaching of vulnerable patients [26]. After cessation of the nation-wide lockdown in Germany, many kindergartens and schools have re-opened, accompanied by the implementation of basic infection-prevention measures [27]. 

This prompted us to perform a survey to evaluate how German POCs handle kindergarten and school attendance for their patients and how the situation was impacted by the SArS-CoV-2 pandemic.

## Methods

A group of experts in pediatric oncology, psychosocial care and infectious diseases developed an internet-based anonymized survey (Survey Monkey™; San Mateo, USA). Main topics and detailed questions were finalized after repeated rounds of internal discussion in the German Society of Pediatric Oncology and Hematology (GPOH)/German Society for Pediatric Infectious Diseases (DGPI) Working Group on infections in immunocompromised patients. In addition, the survey items were extensively discussed with the Psychosocial Working Group of the GPOH (PSAPOH). 

The survey included questions regarding the POCs and their local organization, and seven clinical-case vignettes with clinical situations (Table 1 (Tab. 1)). In addition to predefined answers, individual text comments were allowed. In total, 51 pediatric oncology centers (POCs) were contacted by e-mail. The survey was released on July 07 and closed on August 08, 2020. 

The POCs were arbitrarily categorized into three different size categories, namely small (≤40 newly diagnosed patients/year), medium (41–75), and large (>75) [28]. Datasets were checked for duplicates from the same center. Multiple answers from different participants from the same center were counted as one positive or one negative answer if concordant. Differing answers were categorized as “conflicting results”. Some of the participants replied to only some of the questions. Therefore, the corresponding number of POCs which answered a particular question, are provided in parenthesis (e.g.; 10/51). In addition, conflicting results are outlined separately.

IBM SPSS Statistics Version 24 (IBM Deutschland GmbH, Ehningen) was used for the statistical analysis. Fisher’s test was used to examine differences between categorical variables. A *P*-level < 0.05 was considered as statistically significant (two-tailed).

Since the survey did not contain individual patient data, participation was voluntary, and the participating oncologist consented to the anonymous analysis, approval by an ethics committee was not necessary.

## Results

Forty-one pediatric oncologists from 36 GPOH-affiliated POCs participated in the survey (response rate 70.6% relative to the contacted POCs). The sizes of the participating POCs were equally distributed [small (n=12; 35%), medium (n=12; 35%) and large (n=10; 29%); missing and conflicting reply one each]. Overall, no significant correlation to the center size was seen for any of the items (data not shown).

A total of 91% of all participating POCs provide an in-hospital school/teaching service (missing n=1). Seventeen percent of the POCs have a written standard regarding the (re-)attendance of kindergarten or school, whereas 24% have no such document or official policy, and 59% of the POCs state that they follow a consented good clinical practice approach which, however, has not been codified in a standard document (missing and conflicting replies in 1 and 6 cases, respectively).

Prior to the pandemic, the majority of oncologists stated that the department’s team of medical and psychosocial care providers did not debate about kindergarten or school attendance (91%, n=30, missing and conflicting replies in 1 and 2 cases, respectively). This proportion decreased during the pandemic to 63% (missing replies in one case, no conflicts), which is significantly lower compared to the results prior to the pandemic (*P*=0.0087). Similarly, the proportion of pediatric oncologists who found that home schooling practice was satisfactory was significantly higher prior to than during the pandemic [79% (n=26; missing and conflicting replies one each prior to the pandemic) versus 38% (n=11; missing and conflicting replies in 4 and 3 cases, respectively, during the pandemic); *P*=0.0007].

The replies to the 7 case vignettes are shown in Table 2 (Tab. 2). Out of all pediatric oncologists, 94% (missing and conflicting replies in 3 and 1 cases, respectively) stated that they would appreciate an official recommendation of the GPOH/PSAPOH regarding school or kindergarten attendance for the particular subgroup of patients. 

During the SARS-CoV-2 pandemic, the risk of a SARS-CoV-2 infection in an individual patient was the most important argument against attending school or kindergarten [65% (n=17); missing and conflicting replies in 9 and 1 cases, respectively]. The risk of transmission of the infection from the patient to other patients or caregivers was the most important argument against attending school for only one POC (4%), whereas individual risk and risk of transmission were rated as equally important by 31% (n=8). 

In addition, in the individual text section, team discussions about kindergarten- or school attendance not only focused on the immunocompromised patient, but also on their siblings (same household), who might serve as index persons for SARS-CoV-2 transmission into the family. 

The majority of pediatric oncologists (81%, conflicting replies in 4 cases) stated that the strict restrictions on attending school would result in serious negative psychosocial consequences, whereas 19% of the oncologists stated that negative consequences would be present but not serious. For none of the participants were the restrictions not associated with negative psychosocial consequences for patients and their families.

### Testing for SARS-CoV-2

At the time of the survey, 69% of all participating POCS (n=35, missing n=1) tested all inpatients upon admission, irrespective of symptoms of SARS-CoV-2, whereas two POCs performed SARS-CoV-2 testing only in symptomatic inpatients (e.g., fever, common cold symptoms, cough). 

## Discussion

In-hospital education and home schooling provide pediatric cancer patients and their families a sense of normal life and connection to the “outside world” during intensive treatment [8]. This is important, as patients fear to lose their relationships with friends, classmates and teachers, seek peer acceptance, and struggle to keep up with educational standards according to their age group [12]. As soon as intensive treatment is successfully accomplished, early re-attendance at kindergarten or school is an important step of re-integration into a “normal life” [10], [11]. This also applies to patients who still need outpatient maintenance chemotherapy.

Providing in-hospital education and home schooling by qualified teaching personnel is a common standard in German POCs for children receiving intensive chemotherapy. Interestingly, the survey did not reveal any significant differences between POCs of different sizes (small versus medium versus large). Before the pandemic, the number of home-schooling hours approved by the supervisory school authority of the given federal state for an individual patient was 4 to 6 hours per week (range, 2 hours to 15 hours per week) in most POCs, but is most likely significantly lower now during the pandemic due to the reduced availability of qualified teaching personnel. 

This might also affect the allocation of a dedicated, qualified person to accompany patients with certain disabilities (e.g., related to malignant tumors of the central nervous system and their treatment) in order to facilitate re-attendance in normal education. It is important to note that – according to our survey – there was less consensus within the team of caregivers during the pandemic regarding attendance at kindergarten and school, although the majority of pediatric oncologists agreed that these restrictions will have serious negative psychosocial consequences.

In addition to the limitation of resources of teaching personnel, the concern that the child will be infected with SARS-CoV-2 by unprotected contact with classmates and teachers poses important, difficult problems for attendance at school and kindergarten. This negative impact may extend to healthy siblings, if parents decide to keep them at home in order to protect the patient [23], [24]. In this regard, patients and families need continuous information and education about infection risks and prevention [22]. Restriction of attendance at kindergarten and school might be reasonable during the period of intensive treatment, as early detection of the infection is difficult [18], a fact which, on the other hand, mandates in-hospital schooling and home schooling for these vulnerable patients. Importantly, two-thirds of the POCs perform regular SARS-CoV-2 testing (and quarantine or isolation until return of results) prior to admittance to the ward, irrespective of any symptoms, in order to protect other children undergoing cancer treatment or members of the pediatric oncology treatment team. The reluctance of teachers is therefore not rational, as additional hand disinfection, distancing (≥1.5m), wearing a medical mask [29], and keeping inpatient rooms adequately ventilated with fresh air further reduce the risk of transmission [30]. 

From the perspective of infection prevention, most patients with hematopoietic recovery can re-attend school or kindergarten after completing intensive treatment. In order to reopen kindergartens and schools, particular preventive strategies had to be implemented which were complied with official recommendations from local and federal health authorities [27]. Unfortunately, due to these local specifics, general recommendations by the GPOH are difficult to make, in particular as the pandemic situation is changing constantly [31].

In summary, both in-hospital school education and home schooling of pediatric cancer patients during intensive chemotherapy and re-attendance at school and kindergarten after intensive chemotherapy have suffered a severe setback during the SARS-CoV-2 pandemic. 

This setback is due to several factors, which include fewer available teachers and the fear of infection with SARS-CoV-2. To deal with the latter, continuous communication and education [22] concerning protective measures [29], [30] is required for all medical professionals, patients and their families, as well as caregivers and teachers in order to avoid the detrimental exclusion of pediatric oncology patients from kindergarten and school. 

## Notes

### Competing interests

The German Society for Pediatric Infectious Diseases (DGPI) supported the realization of this survey. AS is second chairperson of the DGPI. MS is the chairman of the German Society for Pediatric Oncology and Hematology (GPOH). TL is the coordinator, and AS, AHG and HJL are members of the Working group on Infections in Pediatric Cancer Patients of the GPOH. AHG: no COIs in the context of this work. MS: no COIs in the context of this work.

### Acknowledgement 

We thankfully acknowledge the fruitful discussions with Iris Lein-Köhler concerning the psychosocial issues of pediatric cancer supportive care, the support of Jana Schmitt with the descriptive analysis, and the statistical analysis by Stanislaw Schmidt.

## Figures and Tables

**Table 1:**
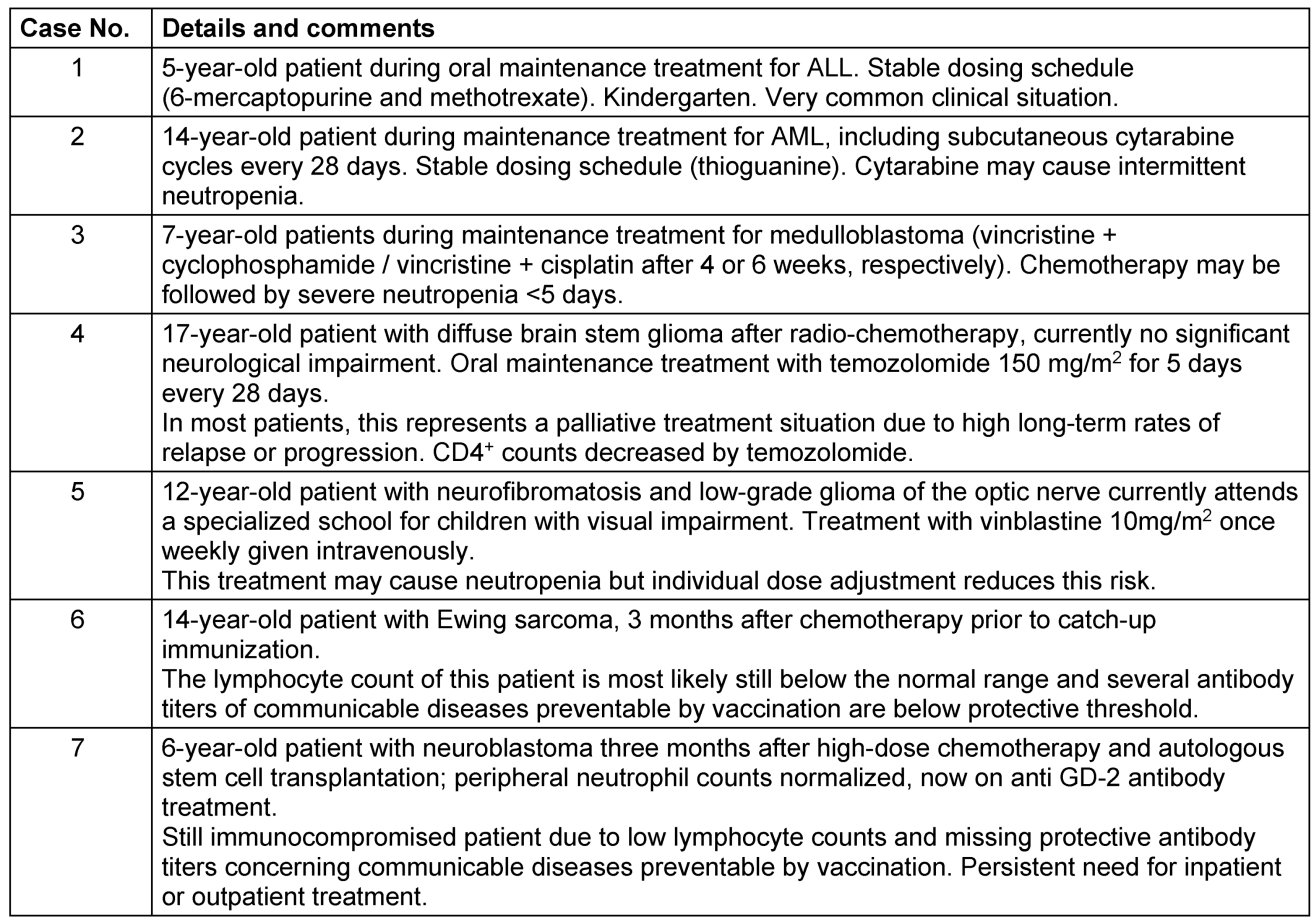
Case vignettes to evaluate the common practice of school and kindergarten attendance before and during the SARS-CoV-2 pandemic

**Table 2:**
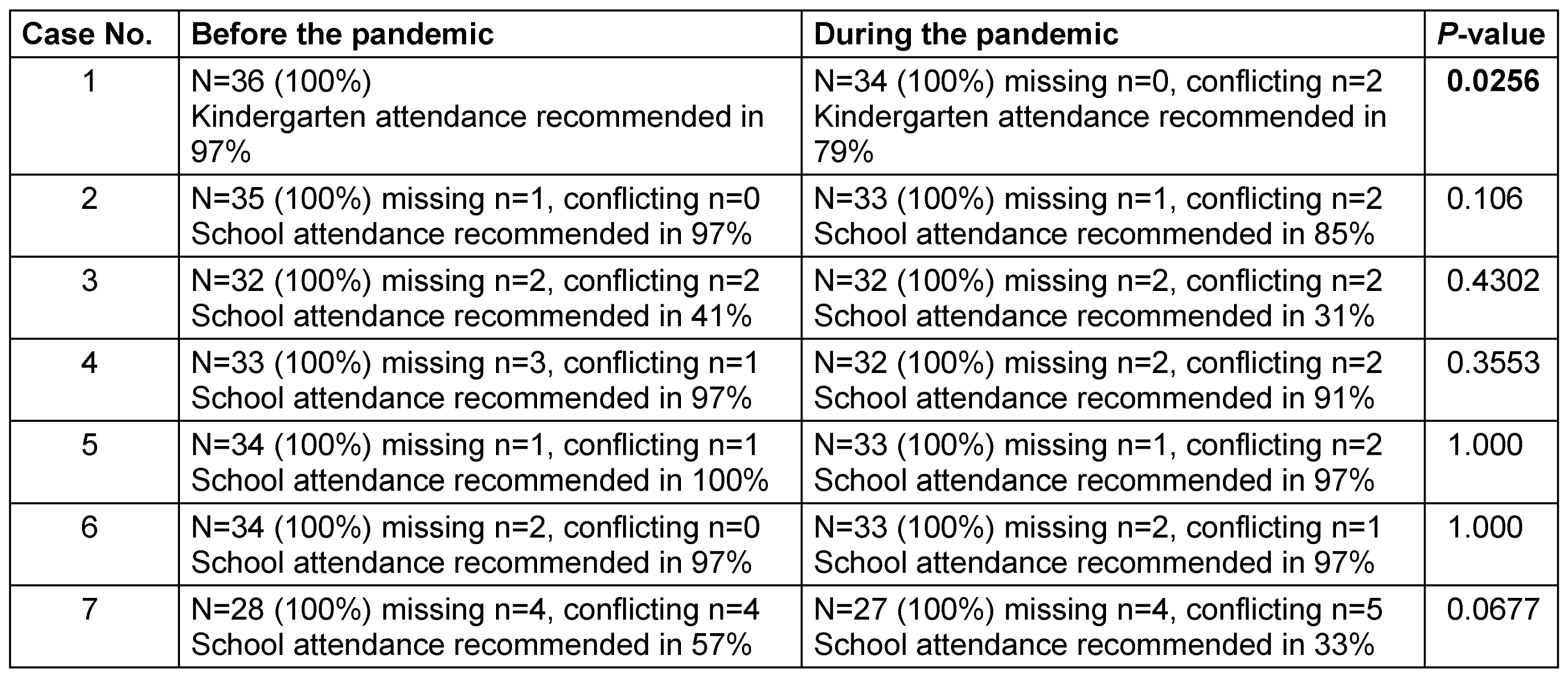
Distribution of the answers concerning the case vignettes; missing values and conflicting results outlined
